# Protocols for Assessing Storage Stability and Solubility of Released N‐Linked Glycans Prior to Mass Spectrometry Analysis

**DOI:** 10.1002/cpz1.70509

**Published:** 2026-10-31

**Authors:** Oluwaseun O. Ajayi, Tunde A. Aduloju

**Affiliations:** ^1^ Department of Chemistry, Franklin College of Arts and Sciences University of Georgia Athens Georgia USA; ^2^ Department of Applied Biochemistry Nnamdi Azikiwe University Awka Nigeria

**Keywords:** freeze–thaw stability, glycomics, LC–IM–MS, N‐linked glycans, sample storage

## Abstract

Released N‐linked glycans are frequently stored between enzymatic release, cleanup, and LC–MS analysis, yet storage solvent and freeze–thaw handling are often reported incompletely or omitted. This unit provides a procedure for determining, within a given laboratory and for a given glycan preparation, whether a selected storage condition preserves analytical performance prior to LC–ion mobility–mass spectrometry (LC–IM–MS). The workflow compares matched aliquots prepared from a single cleared glycan stock, using extracted ion chromatogram signal intensity, chromatographic peak shape, visible precipitation, and replicate precision as acceptance criteria, assessed both before and after a controlled freeze–thaw cycle. A worked example comparing 0.1% (v/v) formic acid in water with 50:50 acetonitrile:water is presented to demonstrate the workflow. The aqueous condition retained LC–IM–MS signal after one freeze–thaw cycle, while the acetonitrile‐containing condition showed substantial post‐thaw signal loss despite acceptable pre‐freeze performance. This example illustrates the magnitude of effect that storage solvent can produce and is not presented as a general storage recommendation, since susceptibility is both structure dependent and platform dependent. The protocols are intended for laboratories analyzing released N‐linked glycans from glycoproteins or biological samples and can be applied to PGC‐, reversed‐phase‐, or HILIC‐based LC–MS workflows, with solvent adjustment performed immediately before injection rather than during long‐term storage. © 2026 The Author(s). *Current Protocols* published by Wiley Periodicals LLC.

**Basic Protocol 1**: Solvent selection and solubility assessment of released N‐linked glycans before LC–IM–MS analysis

**Basic Protocol 2**: Freeze–thaw stability evaluation and long‐term aqueous storage of released N‐linked glycan stocks

**Support Protocol**: Preparation of single‐use glycan aliquots and pre‐injection quality control

## INTRODUCTION

Protein N‐glycosylation is one of the most prevalent post‐translational modifications in eukaryotic systems. N‐linked glycans attached to asparagine residues within the consensus Asn‐X‐Ser/Thr sequon contribute to protein folding, stability, trafficking, molecular recognition, and immune function. Alterations in N‐glycosylation have been associated with a wide range of biological processes, disease states, and therapeutic protein attributes, making accurate glycan characterization a key component of both basic and applied research (Ajayi, 2024; Varki, 2017). Because glycan biosynthesis is not template‐driven, released N‐linked glycans frequently occur as structurally diverse mixtures containing species that differ in branching, linkage, fucosylation, sialylation, and isomeric configuration.

Mass spectrometry has become a principal analytical platform for glycomics because it provides sensitive detection together with structural information derived from accurate mass measurement, chromatographic separation, and fragmentation analysis (Han & Costello, 2013). Recent advances in liquid chromatography–mass spectrometry (LC–MS) and liquid chromatography–ion mobility–mass spectrometry (LC–IM–MS) have improved the characterization of glycan isomers and complex glycan mixtures. Porous graphitic carbon (PGC), hydrophilic interaction liquid chromatography (HILIC), and related chromatographic approaches are widely used for the separation of released glycans prior to mass spectrometric analysis (Ajayi et al., 2025; Ashwood et al., 2019; Young et al., 2021; Zhou et al., 2017]).

Although analytical instrumentation has advanced, less attention has been directed toward the reproducibility of glycan storage and handling prior to analysis. Storage solvent composition, freeze–thaw exposure, aliquoting strategy, and sample preparation practices can introduce variability that is difficult to distinguish from true analytical or biological differences. In many published glycomics studies, storage conditions are reported inconsistently or omitted altogether, limiting reproducibility across laboratories and analytical platforms.

A practical concern is the use of organic solvent‐containing matrices during glycan storage. Acetonitrile‐rich solvents are commonly employed in chromatographic workflows and are sometimes present in samples following cleanup procedures or solvent exchange. However, the suitability of such solvents for prolonged storage is not routinely evaluated. Kozlik et al. (2020) demonstrated that high‐acetonitrile sample solvents can influence recovery of glycan‐containing analytes through structure‐dependent precipitation phenomena. These observations highlight the importance of experimentally validating storage conditions rather than assuming compatibility based on chromatographic requirements alone.

The data presented in this unit are not offered as a general recommendation for glycan storage. A single comparison performed on one glycan preparation, one chromatographic platform, and one freeze–thaw interval cannot establish conditions that will hold across the structural diversity of released N‐glycans or across the range of analytical workflows in current use. Selective loss of sialylated, highly fucosylated, or large complex species may occur under conditions that appear acceptable when judged by total signal alone, and susceptibility to such loss is structure dependent (Kozlik et al., 2020). The appropriate response to this variability is not a universal storage rule but a defined procedure by which each laboratory can determine whether its chosen storage condition preserves analytical performance for its own standards on its own platform. The protocols described here provide that procedure. The comparison reported in Understanding Results is included as a worked example that demonstrates the workflow and illustrates the magnitude of effect storage solvent can produce; it is not the finding this article advances.

To our knowledge, a standardized protocol framework focused specifically on solvent selection, freeze–thaw evaluation, aliquoting practice, and quality control assessment for released N‐linked glycans prior to mass spectrometry analysis has not been described in the Current Protocols format. The protocols presented here provide a practical workflow for reproducible storage and handling of released N‐linked glycans. Basic Protocol 1 compares aqueous and organic‐containing solvent conditions before freezing and establishes a baseline chromatographic profile. Basic Protocol 2 evaluates freeze–thaw effects and defines criteria for long‐term aqueous storage. The Support Protocol describes single‐use aliquoting and routine quality control procedures intended to improve consistency across analytical sessions.

## CAUTIONS

*CAUTION*: Formic acid is corrosive and volatile. Prepare formic acid solutions in a fume hood or other well‐ventilated area. Wear a laboratory coat, chemical‐resistant gloves, and safety glasses. Avoid inhalation of vapors and avoid skin or eye contact.

*CAUTION*: Acetonitrile is flammable and toxic by inhalation, ingestion, and skin absorption. Keep away from ignition sources. Use only in a fume hood and collect acetonitrile‐containing waste according to institutional chemical‐waste procedures.

*CAUTION*: Follow institutional requirements for handling biological samples, glycoprotein sources, enzymes, sharps, and LC–MS solvents. These protocols do not replace local environmental health and safety procedures.

## STRATEGIC PLANNING

Define the downstream LC–MS method before selecting storage conditions. For PGC or reversed‐phase LC–MS, aqueous glycan stock solutions are compatible with immediate preparation for analysis. For HILIC workflows, the injection solvent often requires higher organic content, but this protocol separates stock storage from injection preparation: store the stock in aqueous solvent and adjust the solvent only immediately before injection.

Plan aliquot number and volume from the expected number of analytical sessions. Each aliquot should contain enough material for the intended injections, a quality‐control injection, and pipetting loss. Avoid preparing aliquots that require refreezing after use.

Use the same original stock volume, final solvent composition, injection volume, LC gradient, ion source settings, acquisition method, and data‐processing parameters when comparing conditions. Solvent effects cannot be interpreted reliably if concentration or instrument conditions change between aliquots.

Create a sample log before beginning. Record glycan source, release method, cleanup method, solvent, concentration or relative amount, tube type, preparation date, freezer temperature, freeze–thaw events, injection sequence, analyst, and benchmark chromatogram file name.

*NOTE*: All protocols involving animals must be reviewed and approved by the appropriate Animal Care and Use Committee and must follow regulations for the care and use of laboratory animals. Appropriate informed consent is necessary for obtaining and use of human study material.

## Basic Protocol 1 SOLVENT SELECTION AND SOLUBILITY ASSESSMENT OF RELEASED N‐LINKED GLYCANS BEFORE LC–IM–MS ANALYSIS

This protocol compares matched aliquots of a released N‐linked glycan stock in aqueous and organic‐containing matrices before freezing. The purpose is to determine whether the storage or injection matrix maintains glycan solubility and analytical response. The primary readouts are total ion chromatogram area, extracted ion chromatogram intensity for representative glycan ions, peak shape, visible precipitation, and replicate precision. Use this protocol for each new glycan preparation before committing the remaining stock to long‐term storage.

### Materials

Released N‐linked glycan stock solution prepared by PNGase F release and cleanup (document glycoprotein or biological source, release date, cleanup method, and approximate concentration or normalized injection amount)
0.1% (v/v) formic acid in water (see recipe)
50:50 acetonitrile: water (v/v; see recipe)
LC–MS grade water
LC–MS grade acetonitrile

Vortex mixer
Benchtop microcentrifuge capable of at least 10,000 × *g*
Low‐binding 1.5‐ml polypropylene microcentrifuge tubes
Adjustable‐volume micropipettes and calibrated low‐retention tips
LC–IM–MS system with an established glycan method
Analytical LC column used for glycan analysis, such as PGC, HILIC, or reversed‐phase column validated in the laboratory.
LC–MS vials with low‐volume inserts
Sample log or electronic laboratory notebook.

#### Prepare matched solvent aliquots

1 Thaw the released N‐linked glycan stock at room temperature until the tube has fully equilibrated to room temperature before opening.
  Do not heat the tube to accelerate thawing. Heating can alter labile glycan structures and introduces a variable that is not part of this comparison.
2 Vortex the stock gently for 5 sec and centrifuge for 2 min at 10,000 × *g*, 25°C. Inspect the tube for turbidity, visible particles, film, or pellet formation.
  Record visible precipitation before any transfer. A visible pellet should not be discarded until the solvent comparison is complete.
3 Using a micropipette, transfer the clear supernatant into a fresh low‐binding tube without disturbing any pellet. Mix the transferred stock by gentle pipetting.
4 Prepare Aliquot A by transferring the required stock volume, typically 2 to 10 µl depending on method sensitivity, to a fresh low‐binding tube and bringing it to the final injection volume with 0.1% (v/v) formic acid in water.
  Record the stock volume and final volume. The same stock volume must be used for organic condition.
5 Prepare Aliquot B by transferring the same stock volume to a second low‐binding tube and adjusting to 50:50 (v/v) acetonitrile:water. Mix by pipetting up and down three times.
  Do not vortex vigorously after adding acetonitrile; foaming and droplet retention can reduce transfer accuracy at low volume.
6 Allow Aliquot B to stand at room temperature for 2 min, and then centrifuge both aliquots for 2 min at 10,000 × *g*, 25°C.
  Precipitation after acetonitrile addition is evidence of poor compatibility with the organic matrix and should be documented before injection.
7 Transfer the supernatant from each aliquot to separate LC–MS vials with low‐volume inserts. Label vials with sample ID, solvent condition, replicate number, and date.
8 Inject Aliquot A and Aliquot B using the same LC–IM–MS method, injection volume, ion source settings, and acquisition parameters.
  If material permits, prepare and inject duplicate aliquots for each condition. A recommended sequence is A1, A2, solvent blank, B1, B2, solvent blank.
9 Extract the total ion chromatogram and representative extracted ion chromatograms for abundant glycan ions. Record peak area, peak height, retention time, peak width, and observed peak shape.
10 Compare Aliquot A and Aliquot B. If Aliquot B shows lower signal, visible precipitation, broadened peaks, split peaks, or poor replicate agreement, avoid organic solvent storage and proceed to Basic Protocol 2 using aqueous stock handling.
  Where the organic condition has not been shown to remain stable after freezing in Basic Protocol 2, use the aqueous condition for routine stock storage and adjust solvent immediately before injection.

## Basic Protocol 2 FREEZE–THAW STABILITY EVALUATION AND LONG‐TERM AQUEOUS STORAGE OF RELEASED N‐LINKED GLYCAN STOCKS

This protocol tests whether glycan signal and chromatographic quality are preserved after freezing. It uses the same solvent comparison established in Basic Protocol 1 and adds one freeze–thaw cycle before the second injection set. The outcome defines the storage condition for the remaining stock and documents whether freeze–thaw exposure changes the analytical profile.

### Additional Materials (also see Basic Protocol 1)

Freezer set to −20°C or −80°C; document actual storage temperature

#### Freeze the stock and prepare post‐thaw aliquots

1 Complete Basic Protocol 1.
2 After completing Basic Protocol 1, transfer the remaining cleared glycan stock to a freshly labeled low‐binding tube, if needed. Record the remaining volume and solvent composition.
3 Freeze the stock at the designated storage temperature. Record date, time, freezer temperature, tube type, and sample location in the log.
  Use −80°C for longer storage when available. If −20°C is used, avoid frost‐free or frequently cycled freezers when possible.
4 At the planned evaluation point, remove the frozen stock and thaw at room temperature until fully liquid. Do not heat.
5 Gently vortex for 5 sec, centrifuge for 2 min at 10,000 × *g*, 25°C, and inspect the solution for turbidity or pellet formation. Record observations.
6 Prepare Aliquot C using the same stock volume and final volume as Aliquot A, with 0.1% (v/v) formic acid in water as the matrix.
7 Prepare Aliquot D using the same stock volume and final volume as Aliquot B, adjusted to 50:50 acetonitrile:water (v/v).
8 Centrifuge Aliquots C and D for 2 min at 10,000 × *g*, 25°C, and transfer supernatants to LC–MS vials.
9 Inject Aliquots C and D using the same LC–IM–MS method, injection volume, source settings, and data‐processing parameters used in Basic Protocol 1.
  A recommended sequence is C1, C2, solvent blank, D1, D2, solvent blank.
10 Compare Aliquot C with Aliquot A and Aliquot D with Aliquot B for total signal, representative glycan ion signal, retention time, peak shape, and replicate precision. Representative duplicate‐injection chromatograms before and after freezing are shown for the aqueous condition (Fig. 1) and the organic condition (Fig. 2).

**Figure 1:**
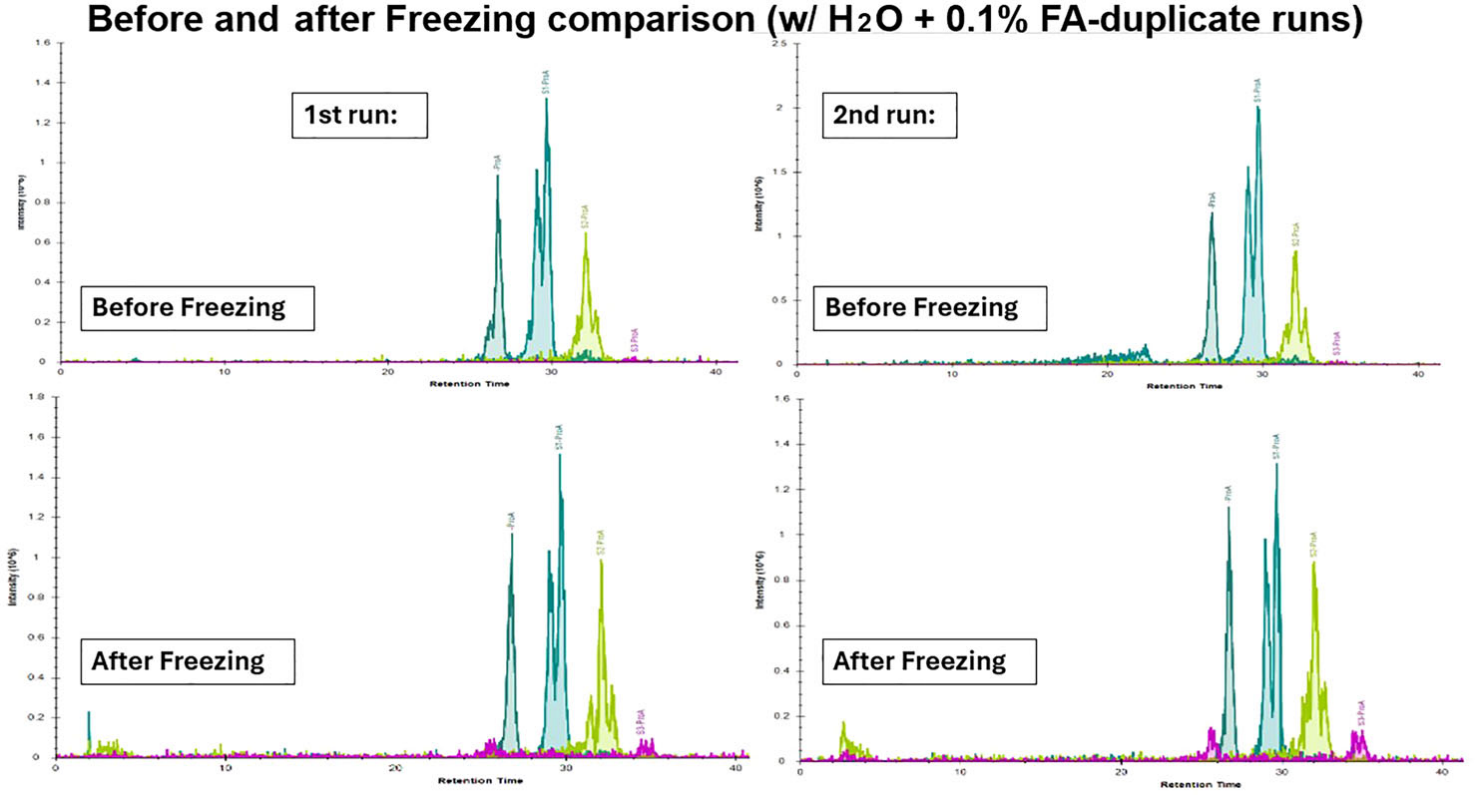
Effect of a single freeze‐thaw cycle on chromatographic performance of released N‐linked glycans stored in 0.1% formic acid in water. Extracted chromatograms (retention time vs. relative intensity) for duplicate injections of the same aqueous glycan stock, acquired before freezing (top panels) and after one freeze‐thaw cycle (bottom panels); left and right panels show two independent replicate injections (1st run, 2nd run). Intensity axes are scaled independently for each panel; comparisons are made within a replicate pair, before versus after freezing, rather than between replicates. Peak retention times, relative peak intensities, and overall chromatographic profile were retained after freezing, indicating that aqueous storage preserved analytical performance under the conditions evaluated.

**Figure 2:**
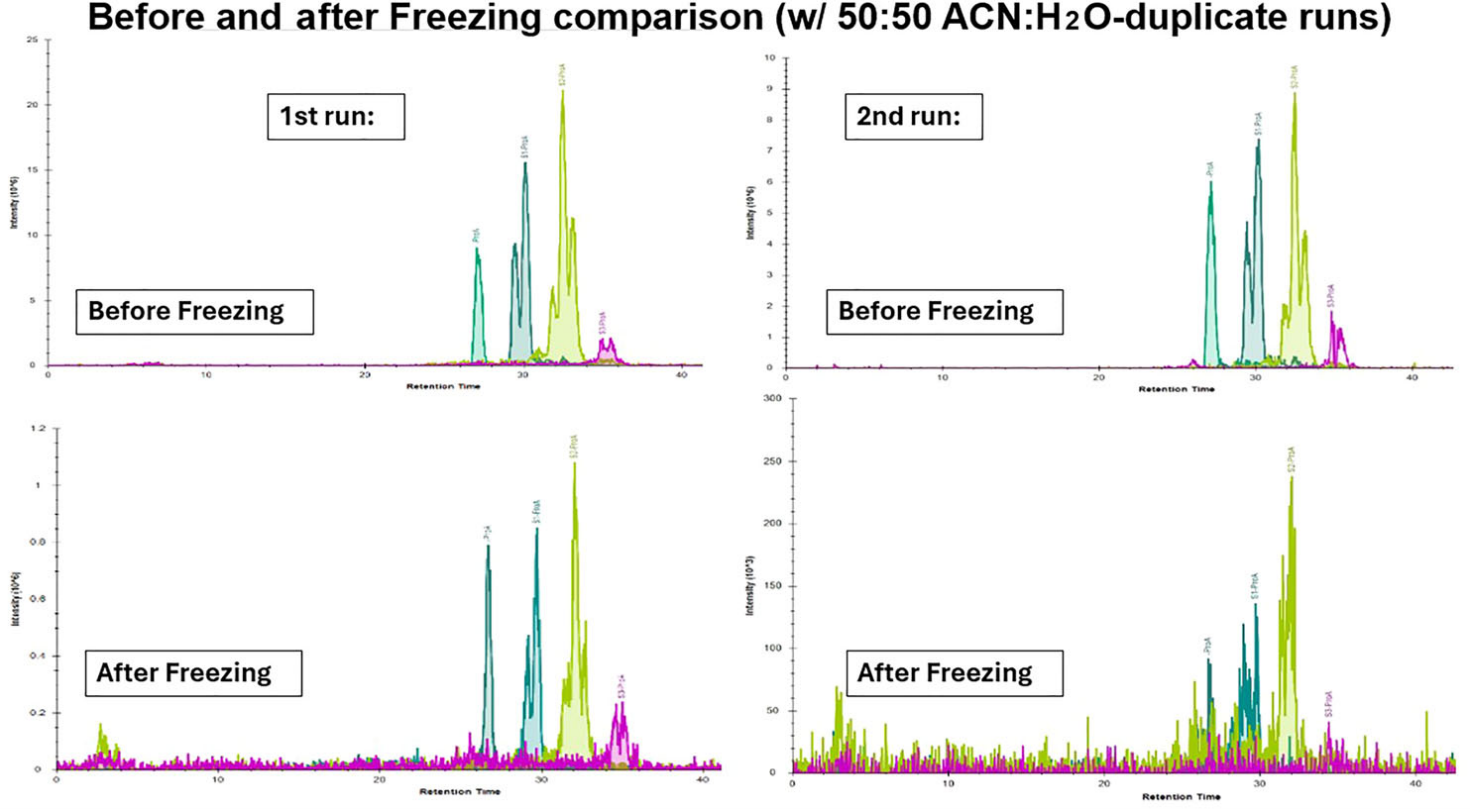
Effect of a single freeze‐thaw cycle on chromatographic performance of released N‐linked glycans stored in 50:50 acetonitrile:water (v/v). Extracted chromatograms for duplicate injections of the same glycan stock in organic‐containing matrix, acquired before freezing (top panels) and after one freeze‐thaw cycle (bottom panels); left and right panels show two independent replicate injections (1st run, 2nd run). Intensity axes are scaled independently for each panel; comparisons are made within a replicate pair, before versus after freezing, rather than between replicates. Peak intensity and chromatographic resolution decreased and baseline noise increased after freezing relative to the matched pre‐freeze injections, indicating loss of analytical performance under organic‐containing storage.

11 Accept aqueous storage if the post‐thaw aqueous chromatogram remains comparable to the pre‐freeze aqueous benchmark and the representative peak areas remain within the laboratory‐defined acceptance window, typically ±15% for duplicate or short‐sequence QC checks.
  If the aqueous sample fails this criterion, do not assume a solvent effect. Recheck injection volume, instrument stability, vial transfer, source condition, and data integration before preparing a new glycan batch.
12 If the organic post‐thaw aliquot shows reduced signal, visible precipitation, or poorer peak quality relative to the organic pre‐freeze aliquot, do not store glycan stocks in 50:50 acetonitrile: water. Store stocks in aqueous solvent and adjust solvent only immediately before injection if the chromatography method requires it.
13 Use the accepted aqueous pre‐freeze or post‐thaw chromatogram as the benchmark for future aliquots from the same stock. Archive the raw file, processed result, extracted ion list, integration method, and sample log entry.

## PREPARATION OF SINGLE‐USE GLYCAN ALIQUOTS AND PRE‐INJECTION QUALITY CONTROL

This support protocol converts an accepted aqueous glycan stock into single‐use aliquots and defines the quality‐control check performed before each analytical session. It is intended to prevent repeated freeze–thaw exposure and to detect loss of recovery before study samples are injected.

### Materials

Aqueous released N‐linked glycan stock (see Basic Protocol 2)

Vortex mixer
Benchtop microcentrifuge capable of at least 10,000 × *g*
Low‐binding 1.5‐ml polypropylene microcentrifuge tubes
Permanent or Cryogenic‐safe labels
Freezer
LC–MS vials with low‐volume inserts
LC–IM–MS system and column used for benchmark analysis.
Sample log or electronic laboratory notebook

1 Thaw the accepted aqueous glycan stock at room temperature, vortex gently for 5 sec, and centrifuge for 2 min at 10,000 × *g*, 25°C.
2 Calculate the aliquot volume needed for one analytical session, including replicate injections, one QC injection, dead volume, and pipetting loss.
  For example, three injections of 5 µl each should not be aliquoted as exactly 15 µl. Prepare at least 20 µl to maintain reliable transfer volume.
3 Dispense the calculated volume into individually labeled low‐binding tubes. Label each tube with glycan source, preparation date, solvent, aliquot number, concentration or normalized amount, and freezer location.
4 Freeze aliquots immediately at the selected storage temperature. Record the number of aliquots prepared and the freezer location.
  Prepare aliquots from a single thawed stock whenever possible to minimize differences among aliquots.
5 Before each analytical session, thaw one aliquot at room temperature, vortex gently, centrifuge for 2 min at 10,000 × *g*, 25°C, and inspect the solution for clarity.
6 Transfer the supernatant to an LC–MS vial and inject one QC run before study samples.
7 Compare the QC chromatogram with the benchmark chromatogram for total ion signal, representative extracted ion signal, retention time alignment, and peak shape.
8 Proceed with the analytical run only if the QC result meets the predefined acceptance criteria. If QC fails, troubleshoot using Table 1 before injecting study samples.

**Table 1:** Summarizes Common Problems Encountered During Solvent Comparison, Freeze–thaw Evaluation, Aliquoting, and LC–IM–MS Injection of Released N‐linked Glycans

Problem	Possible Cause	Solution
Reduced or absent signal after thawing	Glycan precipitation during frozen storage, especially in organic‐containing solvent; poor thaw mixing; transfer loss	Inspect for turbidity or pellet formation; centrifuge and inject the supernatant; compare with benchmark; prepare fresh aqueous aliquots if recovery cannot be restored.
Organic condition acceptable before freezing but poor after freezing	Freeze‐induced loss in 50:50 acetonitrile: water or another high‐organic matrix	Do not store the stock in organic solvent. Store in aqueous solvent and adjust solvent immediately before injection if needed.
High variability between duplicate injections	Unequal stock volume, incomplete mixing, vial insert dead volume, inconsistent injection volume, or partial precipitation	Prepare fresh matched aliquots; verify pipettes and autosampler volume; centrifuge before vial transfer; ensure each vial contains sufficient volume for reliable injection.
Broad, split, or distorted peaks	Injection solvent mismatch, partial precipitation, column overload, or particulate material	Check sample clarity; inject a solvent blank; reduce injection amount if overloaded; adjust injection solvent only according to the validated LC method.
Loss of sialylated glycan signal over storage	Selective loss or degradation of labile glycan species; prolonged warm handling; unsuitable solvent or pH	Minimize room‐temperature exposure; use single‐use aqueous aliquots; compare extracted ion chromatograms for sialylated species against benchmark; prepare a new stock if degradation is confirmed.
New early eluting or unexpected peaks	Carryover, degradation products, contamination, or integration artifacts	Run solvent blanks; review raw spectra and peak integration; inspect solvent and vial cleanliness; compare with a freshly prepared reference when available.
Low recovery from all conditions	Low starting concentration, adsorption to tube or vial surfaces, drying loss, or instrument sensitivity decline	Use low‐binding tubes; avoid unnecessary dry‐down steps; confirm instrument performance with a glycan standard or prior QC sample; increase starting amount if biologically appropriate.
Carryover after organic‐containing samples	Retained material on column or ion source components	Insert solvent blanks; follow the laboratory LC wash procedure; inspect the source if carryover persists; avoid injecting visibly precipitated samples.

9 Discard residual volume from a thawed aliquot after use. Do not refreeze a partially used aliquot.

## REAGENTS AND SOLUTIONS

### Formic acid in water, 0.1% (v/v)

Add 1 mL formic acid (Sigma‐Aldrich, cat. no. 56302 or equivalent) to 999 mL LC‐MS grade water (Fisher Chemical, cat. no. W6‐4, or equivalent) in a clean solvent bottle and mix by inversion.
  Do not heat.
Prepare fresh for each experimental batch or store tightly capped for up to 7 days at room temperature, if permitted by local laboratory practice

### Acetonitrile:water (v/v), 50:50

Combine 50 ml LC‐MS grade acetonitrile (Honeywell, cat. no. 34967 or Fisher Optima, cat. no. A955) and 50 ml LC‐MS grade water (Fisher Chemical, cat. no. W6‐4, or equivalent) in a solvent‐compatible bottle and mix with gentle inversion. Allow the mixture to equilibrate to room temperature before use.
Prepare fresh
  Do not use stored acetonitrile:water mixtures for storage‐comparison experiments.

## COMMENTARY

### Critical Parameters

#### Solvent composition

Solvent composition is the main experimental variable. The purpose of the comparison is not to determine which solvent gives the highest signal on the first injection, but which condition preserves soluble, reproducible glycan recovery after freezing. A high‐organic injection matrix may be useful for a specific LC method, but the stock should not be stored in that matrix unless post‐thaw recovery has been demonstrated.

#### Matched volumes and equivalent sample loading

Aliquots A, B, C, and D must contain the same amount of original glycan stock. Any difference in stock volume, final volume, vial insert recovery, or injection volume will confound the solvent comparison.

#### Centrifugation before injection

Centrifugation removes visible and subvisible particulate material that can alter injection reproducibility and damage LC components. The supernatant should be transferred without disturbing any pellet. If a pellet appears in an organic‐containing aliquot, record it as part of the outcome rather than treating it as routine debris.

#### Freeze–thaw control

The protocol is designed around controlled freeze–thaw exposure. Repeated thawing of a bulk stock can produce cumulative losses and sequence‐dependent variability. Single‐use aliquoting is therefore required once the accepted storage condition has been identified.

#### Benchmark chromatogram

A benchmark chromatogram from the accepted aqueous condition is required for future use of the stock. Keep the raw file, processed method, representative extracted ion list, and integration settings together. A written statement that a chromatogram looked acceptable is not sufficient for reproducible QC.

#### Sample class and concentration

Released glycan mixtures differ in size distribution, sialylation, charge state, and abundance. The acceptance window should be based on the laboratory's LC–MS method and replicate performance. The ±15% criterion used here is a practical QC threshold for duplicate or short‐sequence checks, not a universal statistical rule.

#### When aqueous storage may not be appropriate

Aqueous storage is not universally preferable, and this protocol should not be read as endorsing it by default. Several circumstances argue against it. Prolonged storage of glycans in aqueous solution at low concentration can promote loss through adsorption to container surfaces, so low‐binding or silanized vials may be required. Sialic acid residues are labile and susceptible to hydrolysis, and preparations rich in sialylated species may, therefore, show selective loss during extended aqueous storage that total ion current does not reveal. Aqueous solutions lacking an organic component or preservative are also vulnerable to microbial growth during extended storage above freezing. Finally, laboratories running HILIC separations require high organic content in the injection solvent; this protocol separates stock storage from injection preparation for that reason, but where an organic injection solvent must be prepared in advance, the stability of the prepared sample should itself be evaluated using the workflow described here. These considerations reinforce the central point of the article: the appropriate storage condition is determined empirically for each preparation and platform rather than selected from a published default.

### Sources of Pre‐Analytical Variability

Differences observed during glycan analysis are not always attributable to chromatographic or mass spectrometric performance. Variability may arise during sample storage, solvent exchange, aliquoting, thawing, vial transfer, or injection preparation. Storage solvent composition is of particular importance because analyte recovery can differ between aqueous and organic‐rich environments. Consistent aliquoting practices, controlled freeze–thaw exposure, and routine comparison to a benchmark chromatogram improve the ability to distinguish analytical variation from sample handling artifacts.

### Troubleshooting Table

Refer to Table 1

### Statistical Analysis

For duplicate injections, report individual peak areas and the percent difference or range rather than relying on a formal hypothesis test. For three or more replicate injections, calculating mean, standard deviation, and coefficient of variation for each condition. If the experiment is repeated across independent glycan preparations, analyze normalized peak areas with a model that includes solvent condition and freeze–thaw state as fixed factors and preparation batch as a blocking or random factor. Do not pool different glycan ions unless the normalization strategy and biological rationale are specified in advance.

### Understanding Results

A successful outcome is demonstrated when a glycan preparation maintains comparable chromatographic performance and signal intensity following storage and freeze–thaw handling. In the worked example described here, aliquots stored in 0.1% formic acid in water retained chromatographic profile, peak shape, retention time stability, and overall signal intensity after a single freeze–thaw cycle (Fig. 1). In contrast, aliquots prepared in 50:50 acetonitrile:water exhibited markedly reduced signals and increased baseline noise following freezing, despite acceptable performance prior to storage (Fig. 2). Under the conditions evaluated in this example, the aqueous condition met the acceptance criteria defined in Basic Protocol 1 while the acetonitrile‐containing condition did not. This outcome demonstrates how the workflow discriminates between storage conditions; it does not establish that aqueous storage is appropriate for other glycan preparations, platforms, or storage intervals, each of which must be assessed using the same procedure.

The workflow was subsequently adopted as routine practice in our laboratory and applied across neutral and charged analytes spanning several structural classes: the monosaccharides glucose and galactose, the disaccharide maltose, dextran, a glycopeptide standard, and released N‐glycans from bovine fetuin, human transferrin, and ribonuclease B (Table S1). Aqueous stocks prepared and quality‐controlled according to Basic Protocol 2 and the Support Protocol gave chromatographic profiles consistent with their benchmark chromatograms across repeated analytical sessions, without storage‐related signal loss. Chromatographic data for the bovine fetuin and human transferrin preparations have been reported separately (Ajayi & Ajayi, 2025). These observations reflect routine use of the workflow rather than matched solvent comparisons repeated for each analyte, and indicate that the procedure transfers across analyte classes and across neutral and sialylated species.

When interpreting the chromatograms, examine more than total signal. Representative high mannose, fucosylated, complex, and sialylated glycan ions should be reviewed separately when present. Selective loss of sialylated or larger glycans can distort the apparent glycan profile even if total ion current remains acceptable.

If an aqueous aliquot fails QC, do not immediately conclude chemical degradation. First exclude injection error, autosampler carryover, poor vial recovery, source instability, column issues, incorrect data processing, and integration drift. If the same aliquot continues to fail and a freshly prepared aliquot or stock passes, document the failed aliquot and remove remaining aliquots from use until the storage log is reviewed.

A formal comparative study applying this workflow across glycan classes under matched conditions, including high‐mannose, complex, fucosylated, and sialylated species, and across multiple chromatographic platforms, would establish whether generalizable storage recommendations are achievable for released N‐glycans. Such a study would be a valuable extension of the procedure described here and is a direction the authors encourage.

### Time Considerations

- Basic Protocol 1 requires 30 to 45 min of bench preparation, plus the LC–IM–MS run time required for the selected method and number of injections.
- Basic Protocol 2 requires 20 to 30 min of bench preparation on the post‐thaw day, plus the same LC–IM–MS run time used in Basic Protocol 1. The calendar duration includes at least one freezing interval.
- Τhe Support Protocol requires 20 to 40 min for aliquoting, depending on the number of aliquots, plus one LC–IM–MS QC injection at the beginning of each analytical session.

### Author Contributions

**Oluwaseun O. Ajayi**: Conceptualization; data curation; formal analysis; funding acquisition; investigation; methodology; project administration; resources; software; supervision; validation; visualization; writing—original draft; writing—review and editing. **Tunde A. Aduloju**: Supervision.

### Conflict of Interest

The authors declare no competing financial interests.

## Supporting information




*Supplementary Table S1. Neutral and charged analytes to which the storage and quality‐control workflow has been applied in routine laboratory use*.

## Data Availability

The data that support the findings of this protocol article are available from the corresponding author upon reasonable request, including Raw LC–IM–MS files, processed chromatograms, and sample logs.
