# Supplementary material for: Protocols for Assessing Storage Stability and Solubility of Released N‐Linked Glycans Prior to Mass Spectrometry Analysis
Source: Curr Protoc. 2026 Oct 31;6(11):e70509. doi: 10.1002/cpz1.70509 (PMC13723162; doi:10.1002/cpz1.70509)
Supplement: Supplementary file 1 [file CPZ1-6-0-s001.docx]

**Supporting Information**

**Protocols for Assessing Storage Stability and Solubility of Released N-Linked Glycans Prior to Mass Spectrometry Analysis**

Oluwaseun O. Ajayi and Tunde A. Aduloju

**Table S1.** Neutral and charged analytes to which the storage and quality-control workflow has been applied in routine laboratory use. Aqueous stocks were prepared, aliquoted, and quality-controlled according to Basic Protocol 2 and Support Protocol 1, using 0.1% formic acid in water as the storage solvent throughout. Observations reflect comparison of each session's quality-control injection with the benchmark chromatogram established for that preparation and derive from routine analytical use rather than from matched solvent comparisons repeated for each analyte.

| **Preparation** | **Analyte class** | **Charge** | **Observation** |
| --- | --- | --- | --- |
| Glucose | Monosaccharide | Neutral | Profile consistent with benchmark across repeated analytical sessions |
| Galactose | Monosaccharide | Neutral | Profile consistent with benchmark across repeated analytical sessions |
| Maltose | Disaccharide | Neutral | Profile consistent with benchmark across repeated analytical sessions |
| Dextran | Polysaccharide | Neutral | Profile consistent with benchmark across repeated analytical sessions |
| Glycopeptide standard | Glycopeptide | Neutral | Profile consistent with benchmark across repeated analytical sessions |
| Ribonuclease B | High-mannose N-glycans | Neutral | Profile consistent with benchmark across repeated analytical sessions |
| Bovine fetuin | Sialylated complex N-glycans | Charged | Profile consistent with benchmark across repeated analytical sessions (Ajayi & Ajayi, 2025) |
| Human transferrin | Sialylated complex N-glycans | Charged | Profile consistent with benchmark across repeated analytical sessions (Ajayi & Ajayi, 2025) |

Chromatographic data for the bovine fetuin and human transferrin preparations are reported in Ajayi, O. O., & Ajayi, V. O. (2025), A Systematic Optimization Analysis of N-Linked Glycans on Porous Graphitic Carbon (PGC) at Different Column Temperatures, Journal of Progress in Engineering and Physical Science, 4(6), 22-29, doi:10.63593/JPEPS.2025.12.04.
